# The Sanitary Condition of To-day

**Published:** 1884-09

**Authors:** 


### The Sanitary Condition of To-day.

Dr. Wythe, in the *Pacific M. and S. Jour.*, for March, 1884, says:

"The sanitary condition of the great cities of the world to-day is vastly better than of those of the middle ages, although very far from what common prudence deems desirable. It is still true that people live longer in rural districts than in cities, yet ever since civilization began to inquire into and regulate public hygiene, there has been steady improvement. Statistics may not yet be fully reliable, yet the best we have prove that life has been prolonged by civilization. According to Wagner, 'In the 20th year of life the probable survival was, in the 16th century, 22 years; in this century, 40 years. Among children, the death rate depends upon the social position or high civilization of parents. In England, according to the tables of Mr. Ansell, of 100,000 children born alive, 74,000 will live at the end of their fifth year; but among the upper classes, who can afford the appliances of modern life, there will be 87,000, and among the peerage there will be 90,000.'"

